# Controlled Osteogenic Differentiation of Mouse Mesenchymal Stem Cells by Tetracycline-Controlled Transcriptional Activation of Amelogenin

**DOI:** 10.1371/journal.pone.0145677

**Published:** 2015-12-28

**Authors:** Fangfang Wang, Hiroko Okawa, Yuya Kamano, Kunimichi Niibe, Hiroki Kayashima, Thanaphum Osathanon, Prasit Pavasant, Makio Saeki, Hirofumi Yatani, Hiroshi Egusa

**Affiliations:** 1 Department of Fixed Prosthodontics, Osaka University Graduate School of Dentistry, Suita, Osaka, Japan; 2 Division of Molecular and Regenerative Prosthodontics, Tohoku University Graduate School of Dentistry, Sendai, Miyagi, Japan; 3 Research Unit of Mineralized Tissue, Faculty of Dentistry, Chulalongkorn University, Bangkok, Thailand; 4 Division of Dental Pharmacology, Niigata University Graduate School of Medical and Dental Sciences, Niigata, Japan; University of Texas Southwestern Medical Center, UNITED STATES

## Abstract

Regenerative dental therapies for bone tissues rely on efficient targeting of endogenous and transplanted mesenchymal stem cells (MSCs) to guide bone formation. Amelogenin is the primary component of Emdogain, which is used to regenerate periodontal defects; however, the mechanisms underlying the therapeutic effects on alveolar bone remain unclear. The tetracycline (Tet)-dependent transcriptional regulatory system is a good candidate to investigate distinct roles of genes of interest during stem cell differentiation. Here, we investigated amelogenin-dependent regulation of osteogenesis in MSCs by establishing a Tet-controlled transcriptional activation system. Clonal mouse bone marrow-derived MSCs were lentivirally transduced with the Tet repressor (TetR) expression vector followed by drug selection to obtain MSCs constitutively expressing *TetR* (MSCs-*TetR*). Expression vectors that contained the Tet operator and amelogenin-coding (*Amelx*) cDNA fragments were constructed using the Gateway system and lentivirally introduced into MSCs-*TetR* to generate a Tet regulation system in MSCs (MSCs-*TetR*/*Amelx*). MSCs-*TetR*/*Amelx* significantly overexpressed the *Amelx* gene and protein in the presence of the tetracycline derivative doxycycline. Concomitant expression of *osterix*, *bone sialoprotein* (*BSP*), *osteopontin*, and *osteocalcin* was modulated by addition or removal of doxycycline under osteogenic guidance. During osteogenic induction, MSCs-*TetR*/*Amelx* treated with doxycycline showed significantly increased gene expression of *osterix*, *type I collage*n, *BSP*, and *osteocalcin* in addition to increased alkaline phosphatase activity and mineralized nodule formation. Enhanced extracellular matrix calcification was observed when forced *Amelx* expression commenced at the early stage but not at the intermediate or late stages of osteogenesis. These results suggest that a Tet-controlled *Amelx* gene regulation system for mouse MSCs was successfully established, in which transcriptional activation of *Amelx* was associated with enhanced osteogenic differentiation, especially in the early stage of biomineralization.

## Introduction

After tooth loss, alveolar bone resorption unavoidably occurs; therefore, bone augmentation is often essential in esthetic implant treatment [1]. Mesenchymal stem cells (MSCs), which are multipotent stem cells traditionally found in the bone marrow and subsequently isolated from many other adult tissues, are currently an excellent candidate for regenerative medicine [2]. Recent progress in regenerative approaches using MSCs may provide considerable benefits in dental implant and prosthodontic treatments, facilitating regeneration of atrophic alveolar bone [3]. In addition, activation of MSCs by growth factors is considered to be a promising strategy for efficient oral tissue regeneration [4].

Amelogenin is the primary component of Emdogain, which is used clinically to regenerate periodontal tissues in intrabony defects [5,6]. Amelogenin is secreted by ameloblasts, and it comprises more than 90% of extracellular enamel matrix proteins in developing teeth. During tooth development, amelogenin is known to play a crucial role in the biomineralization and structural organization of enamel [7,8]. Although amelogenin has traditionally been considered an enamel protein, its biological activity in the process of cell differentiation has recently been widely recognized.

Amelogenin is comprised of three domains: an N-terminal tyrosine-rich domain, a central hydrophobic proline-rich domain, and a C-terminal hydrophilic telopeptide [9]. A previous study reported signaling effects of specific amelogenin gene splicing products in cells in an *in vivo* implantation model. These effects included induction of mineralization accompanied by the presence of bone matrix proteins such as bone sialoprotein (BSP, also known as integrin-binding sialoprotein; Ibsp), suggesting a signaling role of amelogenin gene products in preodontoblast maturation [10]. Viswanathan *et al*. demonstrated that recombinant amelogenin regulated *BSP* expression in cementoblasts *in vitro* and also observed a dramatic reduction in the expression of *BSP* in cementoblasts and surrounding osteoblasts in amelogenin-null mice, indicating that amelogenin is a potential regulator of cementum-associated genes [11].

Low levels of amelogenin expression have been reported in non-dental cell types, including stem cells and cells present in bone, brain, and other soft tissues [12], suggesting additional functions of amelogenin such as signal transduction in these cells. Histological observations have shown that amelogenin is expressed at low levels in normal alveolar bone tissues, and that its expression increases at sites of high bone activity and remodeling [13]. In addition, several studies have suggested that amelogenin has a unique function of modulating the osteogenic differentiation of stem cells. In mouse embryonic stem (ES) cells, administration of exogenous leucine-rich amelogenin peptides was demonstrated to rescue a partially amelogenin-null phenotype, and it significantly increased *BSP* and *osterix* expression during osteogenic differentiation [14]. In human bone marrow-derived MSCs, recombinant amelogenin was shown to increase the mRNA level of *alkaline phosphatase* (*ALP*), *type I collagen* and *BSP*, and also to enhance extracellular matrix (ECM) mineralization [15]. Genome-wide expression profiling of *amelogenin*-overexpressing MSCs showed up-regulation of several osteogenesis-associated genes [16]. However, the mechanisms by which amelogenin expression contributes to the osteogenic differentiation of MSCs, particularly the effects of amelogenin expression on mineralization during MSC osteogenesis, remain unclear.

Forced expression of *amelogenin* by lentiviral transduction is one of the most powerful and cost-effective methods to investigate the direct effect of *amelogenin* expression in stem cells [16]. In this system, the viral genome integrates into host chromosomes, and the inserted gene is maintained and expressed in the cells over multiple passages. These properties of lentiviral transduction enable permanent and efficient expression of the transgene in stem cells even after proliferation and differentiation. However, stem cells often lose their intrinsic stemness properties and differentiation capability after viral transduction because of uncontrollable expression of the transgene, thus resulting in heterogeneous populations of cells in different states of differentiation. This drawback makes it difficult to perform reproducible experiments on stem cells using such over-expression systems.

The tetracycline (Tet)-dependent transcriptional regulatory system is one of the best-studied transduction systems with established efficacy of controllable gene expression, where transcription is reversibly turned on or off in the presence or absence of a Tet derivative (doxycycline: Dox) [17]. This system is based on the binding of Dox to the Tet-repressor (TetR) and de-repression of the promoter controlling expression of the gene of interest. The Tet-dependent system has been successfully used to control gene expression in lentiviral transduction systems, including those applied to stem cells [18,19]. Therefore, Tet-inducible gene transcription using a lentiviral transduction system could be a powerful method to investigate and control the functions of amelogenin in MSCs.

In this study, we used on the Tet-dependent lentiviral transcriptional regulatory system to control forced expression of an exogenous *amelogenin* (*Amelx*: *amelogenin*, *X-linked*) gene in MSCs during osteogenic differentiation. The objective of this study was to highlight the *in vitro* mechanisms underlying amelogenin-dependent regulation of osteogenesis in MSCs by establishing a Tet-regulated system for amelogenin expression in MSCs.

## Materials and Methods

### Cell culture

Clonal mouse bone marrow-derived MSCs (mBMSC-4), which we previously established [20], were maintained in the growth medium consisting of modified Eagle minimal essential medium-alpha (α-MEM) (Nacalai Tesque, Kyoto, Japan) supplemented with 15% fetal bovine serum (FBS), 100 units/mL penicillin, 100 μg/mL streptomycin, and 250 ng/mL amphotericin B. For osteogenic induction, cells were cultured in the growth medium supplemented with 0.1 μM dexamethasone, 10 mM β-glycerophosphate, and 50 μM ascorbate-2-phosphate.

Human embryonic kidney (HEK) cell line-derived 293FT cells (Life Technologies, Carlsbad, CA) were maintained in Dulbecco’s modified Eagle medium (DMEM: 4.5 g/L glucose without sodium pyruvate, Nacalai Tesque) with 10% FBS, 0.1 mM MEM non-essential amino acids, 6 mM L-glutamine, 1 mM MEM sodium pyruvate, 50 units/mL penicillin, and 50 μg/mL streptomycin.

### Production of lentiviral vectors carrying *Amelx* gene

The pCMV-SPORT6.1 plasmid vector containing the full-length cDNA of mouse *amelogenin*, *X-linked* (*Amelx*: GenBANK:BC059090.1), was purchased from Open Biosystems (Thermo Scientific). The open reading frame of the *Amelx* cDNA (660 bp) was PCR-amplified (forward primer: CAC CAT GGG GAC CTG GAT TTT GTT; reverse primer: TCA TTT TTC TGT TGT GCT TTC C) and cloned into the pENTR^™^/D-TOPO vector using the pENTR Directional TOPO cloning kit (Life Technologies) to obtain the entry vector (pENTR^™^/D-TOPO/*Amelx*) for the Gateway cloning system (Life Technologies). Using this entry vector, the expression vector for *Amelx* [pLenti6.3/Tet operator (TO)/V5/*Amelx*] was constructed through the LR recombination reaction of the Gateway cloning system.

293FT cells were cultured in 6-cm dishes to produce the lentivirus from the pLenti3.3/*TetR* (Life technologies) and pLenti6.3/TO/V5/*Amelx* expression vectors. The plenti6.3/V5-GW/*green fluorescent protein* (*GFP*) expression vector (Life Technologies) was used as a control vector to examine transduction efficiency. The pLenti3.3/*TetR* vector contains a neomycin resistance gene and the pLenti6.3/TO/V5/*Amelx* and Plenti6.3/V5-GW/*GFP* vectors contain a blasticidin resistance gene for stable selection in mammalian cells. Three micrograms of Virapower Packaging Mix, 1 μg of the expression vector, and 12 μl of Lipofectamine 2000 (Life Technologies) were mixed in 1 ml of OPTI-MEM I (Life Technologies). After 25 minutes of incubation, the mixture was added to the 293FT cells. After 48 hours, the virus-containing supernatant was collected and filtrated with a 0.45-μm cellulose acetate filter.

### Lentiviral-mediated transduction of *TetR* and *Amelx*

**Fig 1:**
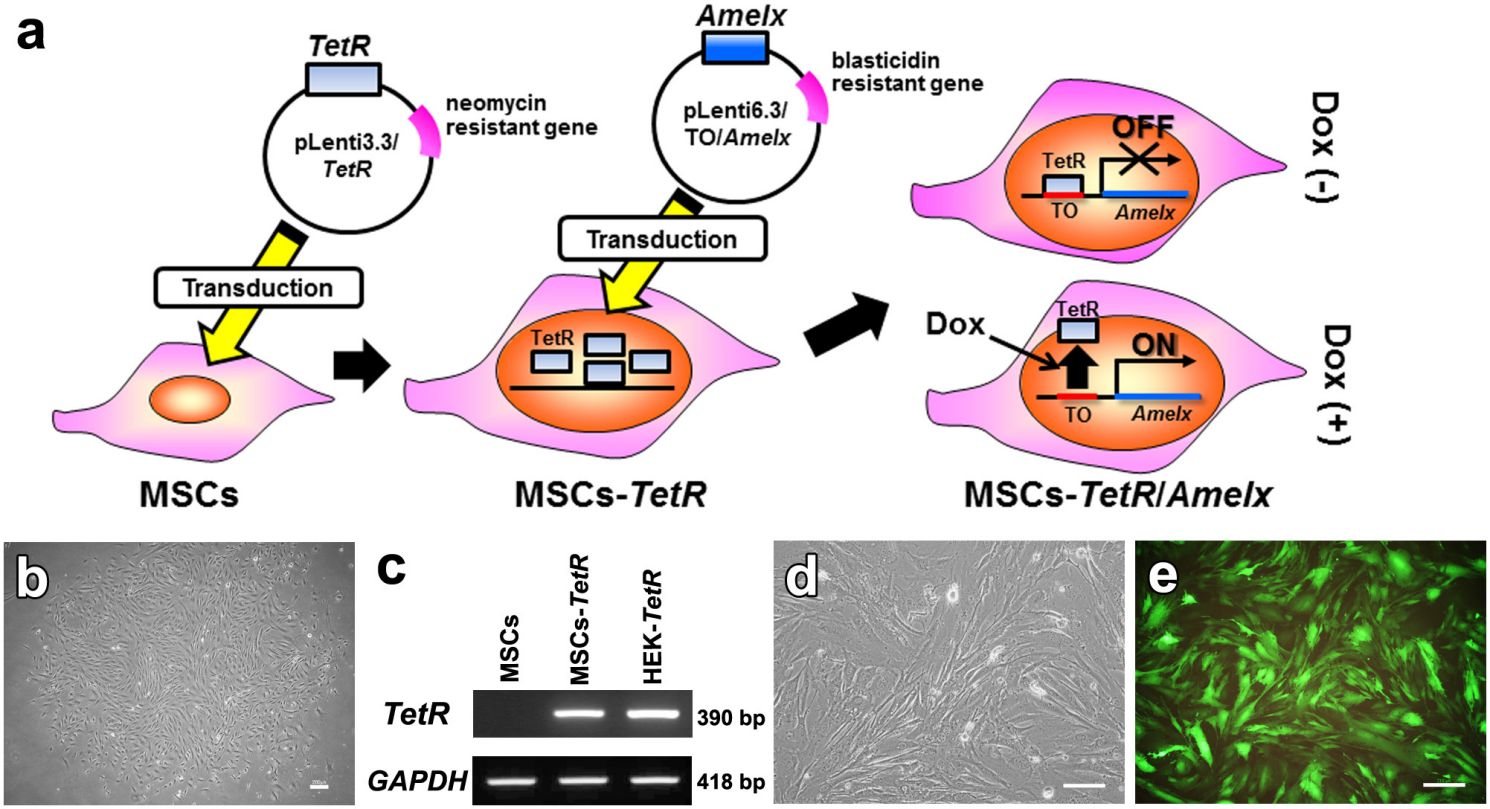
Establishment of a tetracycline (Tet)-controlled *Amelx* expression system in MSCs. (**a**): Schematic diagram depicting the procedure to establish a Tet-controlled *Amelx* expression system in MSCs. TetR: Tet repressor, TO: Tet operator, Dox: doxycycline (tetracycline derivative). (**b**): MSC colony (MSC-*TetR*) in culture medium containing 500 μg/mL geneticin 10 days after transduction with the pLenti3.3/*TetR* expression vector (bar; 60 μm). (**c**): Expression of TetR repressor gene in MSCs (without transduction) and MSCs-*TetR* was determined by RT-PCR. HEK293 cells subjected to the same transduction procedure (HEK-*TetR*) were used as a positive control. (**d, e**): MSCs-*TetR* were lentivirally transduced with the expression vector pLenti6.3/TO/V5/*Amelx* or plenti6.3/V5-GW/*GFP*. MSCs-*TetR*/*Amelx* (**d**) and MSCs-*TetR*/*GFP* (**e**) were selected by 10 μg/mL blastcidin S (bar; 200 μm).

MSCs were cultured in 6-cm dishes in the growth medium. When the cells reached 80% confluence, the medium was replaced with the lentiviral stock solution containing pLenti3.3/*TetR*, and the cells were cultured overnight at 37°C under 5% CO_2_ (Fig 1a). Then, the lentivirus-containing medium was replaced with fresh growth medium. After 3 days, the cells were treated with geneticin (500 μg/ml) (Life Technologies). After 5 days, the surviving cell colonies were picked up to generate MSC clones (MSC-*TetR*) that strongly expressed the *TetR* gene. Expression of *TetR* in MSCs-*TetR* was examined by RT-PCR analysis.

MSCs-*TetR* were seeded at a density of 3×10^5^ cells in a 6-cm dish in the growth medium and incubated overnight at 37°C under 5% CO_2_. Then, the medium was replaced with the viral stock solution of pLenti6.3/TO/V5/*Amelx* or plenti6.3/V5-GW/*GFP* supplemented with 4 μg/ml polybrene (Nacalai Tesque). After 24 hours, the cells were washed once with phosphate-buffered saline (PBS) and cultured in fresh growth medium. After 5 days, the cells were treated with 10 μg/ml blasticidin S (Funakoshi, Tokyo, Japan) to select colonies of MSCs-*TetR* expressing *Amelx* (MSCs-*TetR*/*Amelx*) or *GFP* (MSCs-*TetR*/*GFP*). Tet-dependent expression of the *Amelx* gene in MSCs-*TetR*/*Amelx* in the presence or absence of Dox (2 μg/mL) was evaluated by RT-PCR and western blotting.

### Assessment of osteogenic differentiation of MSCs-*TetR*/*Amelx*

MSCs-*TetR*/*Amelx* were cultured in the growth or osteogenic induction medium in the presence or absence of Dox (2 μg/mL) for 7–35 days. Tet-dependent expression of *Amelx*, osteogenic markers [*Runx2*, *osterix*, *osteocalcin*, *osteopontin* (also known as *secreted phosphoprotein 1*; *Spp1*), *BSP*, and *type I collagen*], and odontoblastic markers [*dentin matrix protein 1* (*DMP1*) and *dentin sialophosphoprotein* (*DSPP*)] was evaluated by semi-quantitative RT-PCR and quantitative real-time RT-PCR. *Glyceraldehyde-3-phosphate dehydrogenase* (*GAPDH*) was used as an internal control.

ALP activity was analyzed by ALP staining. The cells in 24-well plates were washed with PBS and fixed in 10% buffered formalin phosphate for the ALP assay. Cells were stained by incubation with 1.8 mM Fast Red TR (Sigma) and 0.9 mM naphthol AS-MX phosphate (Sigma) in 120 mM Tris buffer (pH 8.4) for 30 min at 37°C [21].

Mineralized nodule formation was evaluated by von Kossa staining. After washing with PBS and fixation with 10% formalin in phosphate buffer, the cells in 24-well plates were placed in 5% AgNO_3_ and exposed to ultraviolet (UV) light for 20 minutes. Subsequently, the cells were washed with distilled water and treated with 5% Na_2_S_2_O_3_ for 5 minutes [22].

Calcium deposition was evaluated by Alizarin Red S staining. After washing with PBS and fixation with 10% formalin in phosphate buffer, the cells were incubated in 40 mM Alizarin Red S (Sigma) in 24-well plates for 20 minutes with gentle shaking. After washing with distilled water, the samples were scanned to obtain digital images. Next, 400 μl of 10% acetic acid was added to the samples, which were then incubated at room temperature for 30 minutes for quantitative analysis [23]. Briefly, the stained samples were collected using a cell scraper and transferred into a 1.5-ml microcentrifuge tube. After heating at 85°C for 10 minutes and cooling on ice for 5 minutes, the samples were centrifuged at 20,000 g for 15 minutes. The colored supernatant (250 μl) was collected into a new tube and 100 μl of 10% ammonia was added to each tube. The optical density of the supernatant sample was measured at 405 nm.

### RT-PCR analysis

RT-PCR analysis was performed as previously described [24]. Total RNA was extracted using an RNeasy Mini Kit (Qiagen, Hilden, Germany). After DNase I treatment (Ambion, Austin, TX), cDNA was synthesized from 1 μg of total RNA using Super Script III reverse transcriptase (Life Technologies). The cDNA target was amplified by PCR using Taq DNA polymerase (Promega, Madison, WI) following the manufacturer’s recommendations. The primer pairs used are described in Table 1. PCR products were subjected to 1.5% agarose gel electrophoresis with ethidium bromide staining and visualized under UV light illumination.

**Table 1:** Primers used for RT-PCR analyses.

Gene	Primers (Fw, forward; Rv, reverse)	Ann Temp^a^	Product size (bp)
*TetR*	Fw: 5’-CGCCTTAGCCATTGGATGT-3’	61°C	390
	Rv: 5’-TCTGCACCTTGGTGATCAAA-3’		
*Amelx*	Fw: 5’-CAGCAACCAATGATGCCAGTTCCT-3’	59.6°C	293
	Rv: 5’-ACTTCTTCCCGCTTGGTCTTGTCT-3’		
*BSP*	Fw: 5’-AAAGTGAAGGAAAGCGACGA-3’	58°C	214
	Rv: 5’-GTTCCTTCTGCACCTGCTTC-3’		
*Runx2*	Fw: 5’-CCGCACGACAACCGCACCAT-3’	65°C	289
	Rv: 5’-CGCTCCGGCCCACAAATCTC-3’		
*osterix*	Fw: 5’-CTTAACCCAGCTCCCTACCC-3’	59°C	270
	Rv: 5’-TGTGAATGGGCTTCTTCCTC-3’		
*osteocalcin*	Fw: 5’-AAGCAGGAGGGCAATAAGGT-3’	60°C	292
	Rv: 5’-AGCTGCTGTGACATCCATAC-3’		
*osteopontin*	Fw: 5’-TCACCATTCGGATGAGTCTG-3’	55°C	437
	Rv: 5’-ACTTGTGGCTCTGATGTTCC-3’		
*GAPDH*	Fw: 5’-CACCATGGAGAAGGCCGGGG-3’	67°C	418
	Rv: 5’-GACGGACACATTGGGGGTAG-3’		

^a^Ann Temp: Annealing temperature

For quantitative real-time PCR analysis, SYBR Green and TaqMan assays were performed using Thunderbird SYBR qPCR Mix (Toyobo, Osaka, Japan) and TaqMan Gene Expression PCR Master Mix (Applied Biosystems, Foster City, USA), respectively, on an Applied Biosystems 7300 real-time PCR system (Applied Biosystems). The primer pairs used are shown in Table 2. Target gene expression was quantitatively analyzed using the ΔΔCt method [25].

**Table 2:** Primers used for quantitative real-time RT-PCR analyses.

Gene	Primers (Fw, forward; Rv, reverse)	Ann Temp^a^	Product size (bp)
*osterix*	Fw: 5’-CTCGTCTGACTGCCTGCCTAG-3’	60°C	84
	Rv: 5’-GCGTGGATGCCTGCCTTGTA-3’		
*Collagen 1a1*	Fw: 5’-TGTCCCAACCCCCAAAGAC-3’	60°C	92
	Rv: 5’-CCCTCGACTCCTACATCTTCTGA-3’		
*osteocalcin*	Fw: 5’-CCGGGAGCAGTGTGAGCTTA-3’	60°C	68
	Rv: 5’-AGGCGGTCTTCAAGCCATACT-3’		
*GAPDH*	Fw: 5’-TGCACCACCAACTGCTTAG-3’	60°C	177
	Rv: 5’-GGATGCAGGGATGATGTTC-3’		
*Runx2*	Mm00501578_m1*		
*BSP*	Mm00492555_m1*		
*osteopontin*	Mm00436767_m1*		
*Amelx*	Mm01166221_m1*		
*DMP1*	Mm01208363_m1*		
*DSPP*	Mm00515666_m1*		
*GAPDH*	NM_008084/ Mm99999915_g1*		

^a^Ann Temp: Annealing temperature

*PCR primers and a TaqMan probe (Applied Biosystem) for the TaqMan assay

### Western blotting analysis

Cell pellets were collected when the cells reached 90% confluence, and the cells were lysed with RIPA buffer [50 mM Tris-HCl, pH 8.0, 150 mM NaCl, 1% Nonidet P-40 (NP-40), 1% sodium deoxycholate, and 0.1% SDS] supplemented with protease inhibitor cocktail (Nacalai Tesque). Proteins from the cell lysates were separated by 10% SDS-polyacrylamide gel electrophoresis and transferred to a polyvinylidine difluoride membrane (Millipore). The blots were blocked with TBST (10 mM Tris-HCl, pH 7.4, 100 mM NaCl, and 0.1% Tween-20) containing 5% skim milk, and then incubated with primary monoclonal antibodies against amelogenin (1:1000; clone F11, Santa Cruz Biotechnology, Santa Cruz, CA) or GAPDH (1:10000, clone 6C5, Millipore) at 4°C overnight. After washing with TBST, the membrane was incubated with anti-mouse IgG HRP-linked antibody (1:3000, Cell Signaling) for 1 hour at room temperature. Signals were detected using Immobilon Western Chemiluminescent HRP Substrate (Millipore) and the FPM 100 imaging system (Fujifilm, Tokyo, Japan).

### Statistical analyses

One-way analysis of variance (ANOVA) with Tukey’s post hoc test was used for comparison of more than 2 groups. A significant difference was defined when *P* < 0.05.

## Results

### Establishment of a Tet-controlled *Amelx* expression system in MSCs

Ten days after the transduction of MSCs with the pLenti3.3/*TetR* expression vector, few colonies were observed in the culture containing 500 μg/mL geneticin (Fig 1b). The colonies were picked up and clonal cultures of MSCs (MSCs-*TetR*) were established. RT-PCR showed that MSCs-*TetR* strongly expressed *TetR* mRNA at levels comparable to those in control *TetR*-expressing HEK293 cells subjected to the same transduction procedure (Fig 1c).

The pLenti6.3/TO/V5/*Amelx* expression vector was lentivirally introduced into the MSCs-*TetR* to generate a Tet-controlled *Amelx* expression system. The pLenti6.3/V5-GW/*GFP* expression vector was also separately introduced into MSCs-*TetR* to determine the percentage of transduced cells in the population of surviving cells after drug selection. Seven days after the transduction of MSCs-*TetR* with each expression vector and culture in Blasticidin S, the surviving colonies were collected and referred to as MSCs-*TetR*/*Amelx* (Fig 1d) or MSCs-*TetR*/*GFP*. The percentage of transduced cells in the populations of MSCs-*TetR*/*Amelx* was estimated as >90%, as determined by *GFP* expression in MSCs-*TetR*/*GFP* (Fig 1e).

### Induction of *Amelx* expression in MSCs-*TetR*/*Amelx* by Dox treatment

**Fig 2:**
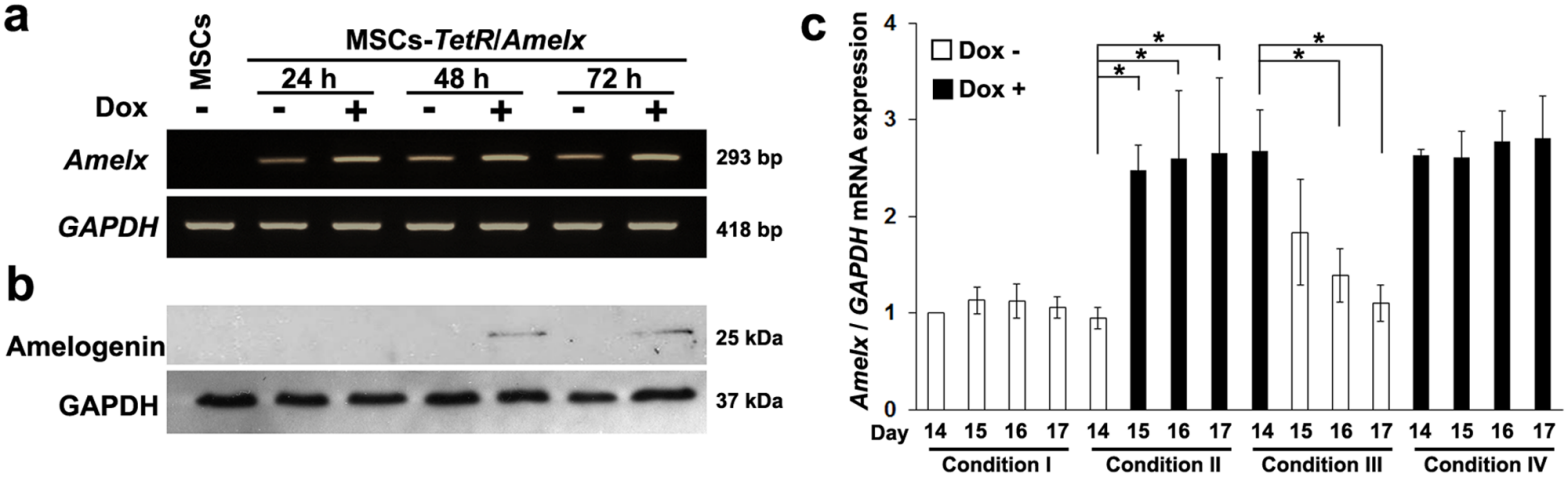
Induction of *Amelx* expression in MSCs-*TetR*/*Amelx* by Dox treatment. MSCs-*TetR*/*Amelx* were cultured in the growth medium in the presence (**+**) or absence (**-**) of Dox for 24–72 hours. Inducible expression of the *Amelx* gene was detected by RT-PCR (**a**) and western blot (**b**) analyses. GAPDH was used as a loading control. (**c**) MSCs-*TetR*/*Amelx* were cultured in the osteogenic induction medium in the presence (+) (black bars) or absence (-) (white bars) of Dox for 17 days in four different conditions. (Condition I: day 0–17 Dox-; Condition II: day 0–14 Dox-, day 15–17 Dox+; Condition III: day 0–14 Dox+, day 15–17 Dox-; Condition IV: day 0–17 Dox+). Expression of *Amelx* was determined by quantitative real-time RT-PCR analysis. Significant differences (*P<0.01: ANOVA with Tukey’s multiple comparison test: n = 4) within each condition are shown.

When MSCs-*TetR*/*Amelx* were cultured in the presence of Dox, enhanced gene expression of *Amelx* was confirmed after 24 hours (Fig 2a). Western blotting analysis showed that Dox stimulated MSCs-*TetR*/*Amelx* to express amelogenin protein after 48 hours (Fig 2b).

MSCs-*TetR*/*Amelx* were cultured in osteogenic induction medium for 17 days in four different conditions, i.e., (I) in the absence of Dox for 17 days; (II) in the absence of Dox until day 14 and then in the presence of Dox from day 15 to 17; (III) in the presence of Dox until day 14 and then in the absence of Dox from day 15 to 17; and (IV) in the presence of Dox for 17 days. Quantitative real-time RT-PCR showed that expression of *Amelx* in cells from day 14 to 17 in Condition IV was markedly higher than that in Condition I (Fig 2c). In Condition II, expression of *Amelx* significantly increased on day 15 (one day after Dox addition). In contrast, in Condition III, expression of *Amelx* markedly decreased after day 15 (one day after Dox depletion), and significantly decreased after day 16.

**Fig 3:**
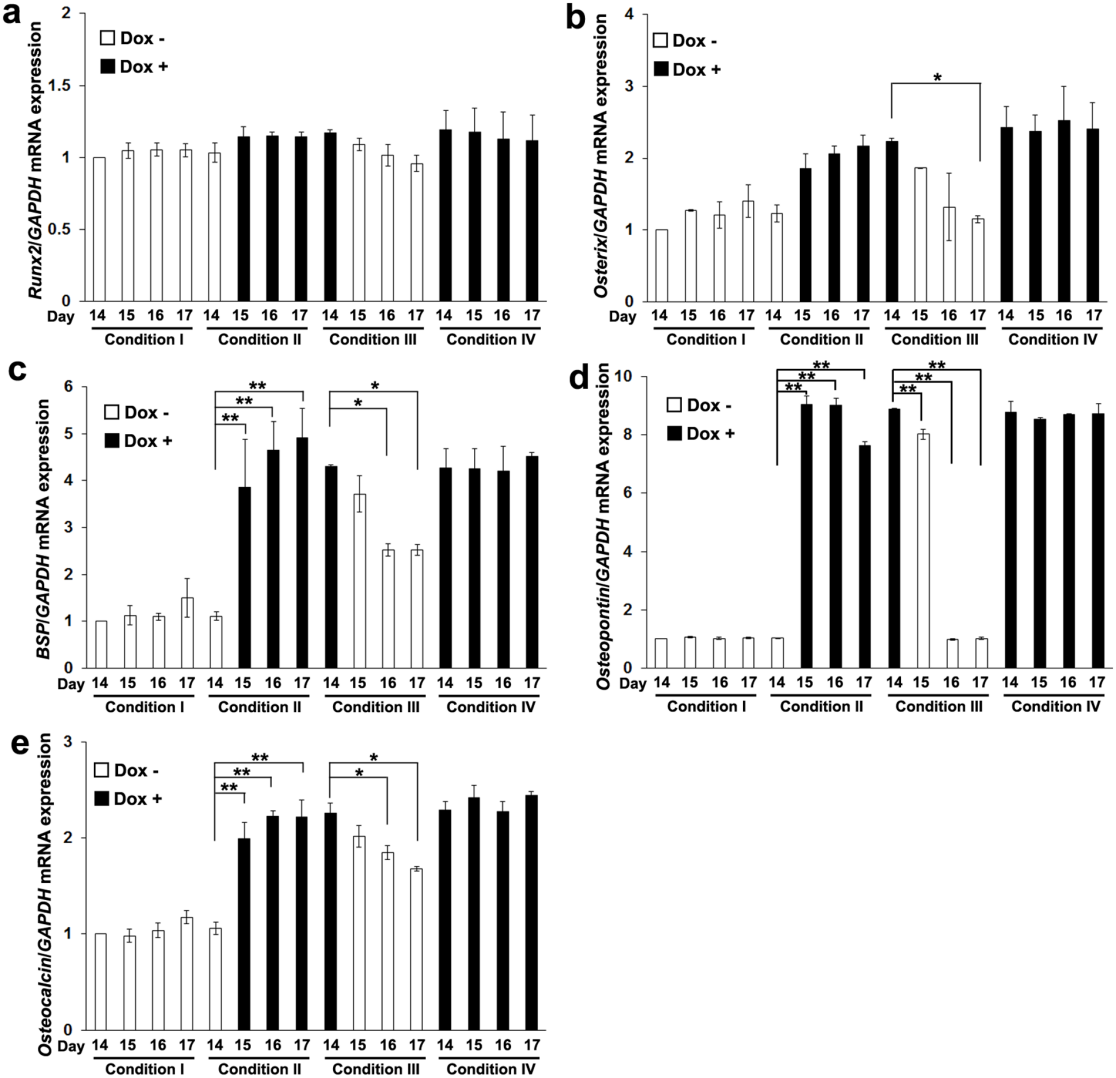
Controllable expression of *osterix*, *BSP* and *osteocalcin* in MSCs-*TetR*/*Amelx* by Dox treatment. MSCs-*TetR*/*Amelx* were cultured in the osteogenic induction medium in the presence (+) (black bars) or absence (-) (white bars) of Dox for 17 days in four different conditions. (Condition I: day 0–17 Dox-; Condition II: day 0–14 Dox-, day 15–17 Dox+; Condition III: day 0–14 Dox+, day 15–17 Dox-; Condition IV: day 0–17 Dox+). Expression of *Runx2* (**a**), *osterix* (**b**), *BSP* (**c**), *osteopontin* (**d**) and *osteocalcin* (**e**) was determined by a quantitative real-time RT-PCR analysis. Significant differences (**P<0.01, *P<0.05: ANOVA with Tukey’s multiple comparison test: n = 3) within each condition are shown.

In these conditions, *Runx2* expression was not significantly altered (Fig 3a). In contrast, expression of *osterix* was significantly decreased by the Dox depletion (Condition III) on day 17 (Fig 3b). Expression of *BSP* (Fig 3c), *osteopontin* (Fig 3d), and *osteocalcin* (Fig 3e) was significantly enhanced by addition of Dox (Condition II) and decreased by Dox depletion (Condition III).

### Effects of forced expression of *Amelx* in MSCs-*tetR*/*Amelx* on osteogenic differentiation

**Fig 4:**
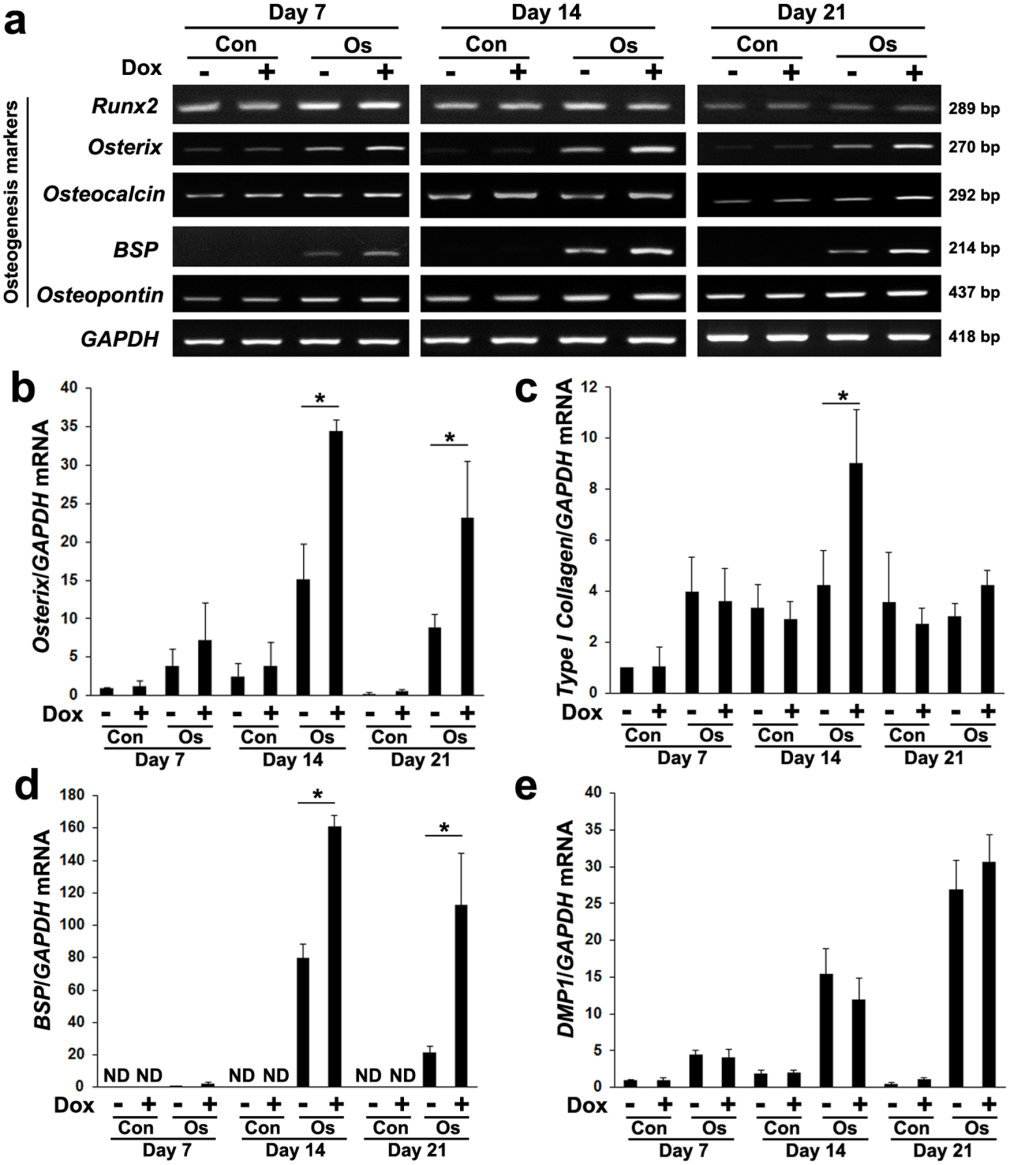
Effects of forced expression of *Amelx* in MSCs-*tetR*/*Amelx* on expression of osteogenic marker genes. MSCs-*TetR*/*Amelx* were cultured in growth medium (Con) or osteogenic induction medium (Os) in the presence (**+**) or absence (-) of Dox for 21 days. (**a**) The expression of osteogenic marker genes (*Runx2*, *osterix*, *osteocalcin*, *BSP*, and *osteopontin*) was examined by RT-PCR analysis. *GAPDH* was used as an internal control. Quantitative real-time RT-PCR analysis was performed to examine expression of *osterix* (**b**), *type I collagen* (**c**), *BSP* (**d**), and *DMP1* (**e**) genes. *GAPDH* was used as an internal control. Significant differences (*P<0.01: ANOVA with Tukey’s multiple comparison test: n = 3) are shown.

When MSCs-*TetR*/*Amelx* were cultured in osteogenic induction medium with Dox, enhanced expression of *osterix*, *osteocalcin*, and *BSP* genes was observed (Fig 4a). In contrast, expression of *Runx2* was not significantly altered by the addition of Dox. Quantitative real-time RT-PCR demonstrated Dox-induced expression of *osterix* (Fig 4b), *type I collagen* (Fig 4c), and *BSP* (Fig 4d) genes after 14 days of osteogenic induction. Dox did not significantly alter the expression of *DMP1* in MSCs-*TetR*/*Amelx* in the growth medium or in the osteogenic induction medium (Fig 4e). Expression of *DSPP* was not detected in MSCs-*TetR*/*Amelx* under any culture condition.

**Fig 5:**
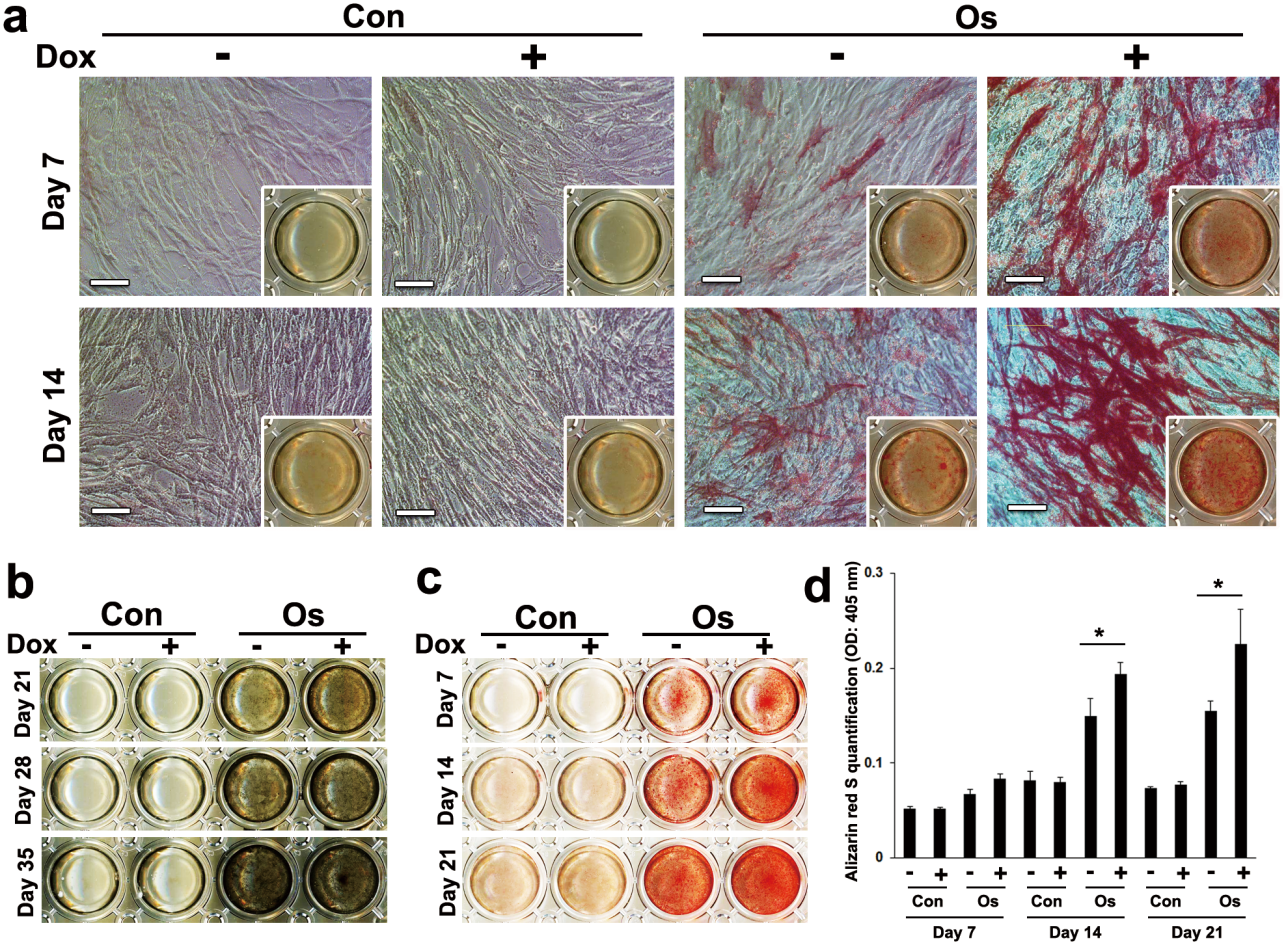
Effects of forced expression of *Amelx* on ALP activity and mineralized nodule formation. MSCs-*TetR*/*Amelx* at 3 passages were cultured in the growth medium (Con) or osteogenic induction medium (Os) in the presence (**+**) or absence (-) of Dox in 24-well plates. (**a**) ALP activity on day 7 and 14 was examined by ALP staining (bars: 100 μm). (**b**) Mineralized nodule formation on day 21, 28 and 35 was detected by von Kossa staining. (**c, d**): Calcium deposition was determined by Alizarin Red S staining on day 7, 14 and 21 (**c**) and the staining intensity was quantitatively analyzed (**d**). Significant differences (*P<0.01: ANOVA with Tukey’s multiple comparison test: n = 9) are shown.

Dox induced ALP activity (Fig 5a) and mineralized nodule formation (Fig 5b) of MSCs-*TetR*/*Amelx* in osteogenic induction medium after 7 and 21 days, respectively. Alizarin Red S staining also supported the enhancement of MSC-*TetR*/*Amelx* matrix calcification by Dox treatment (Fig 5c). Quantitative analysis of Alizarin Red S staining showed that Dox treatment significantly induced calcium deposition of MSCs-*TetR*/*Amelx* after 14 days in the osteogenic induction medium (Fig 5d).

### Effects of forced expression of *Amelx* on matrix calcification at different osteogenic differentiation stages

**Fig 6:**
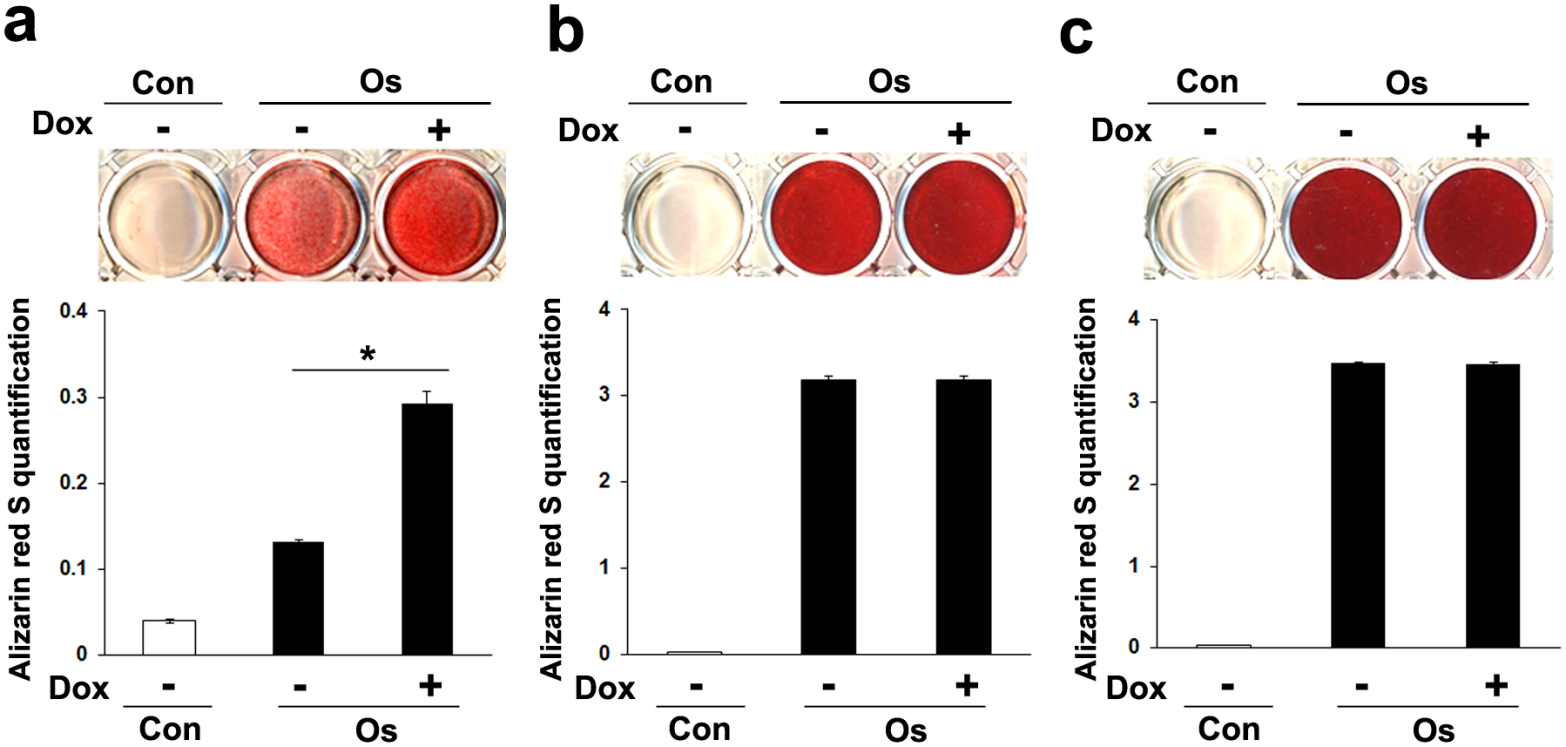
Effects of forced expression of *Amelx* at different osteogenic differentiation stages on matrix calcification of MSCs. MSCs-*TetR*/*Amelx* at 8 passages were cultured in the growth medium (Con) or osteogenic induction medium (Os). Dox was applied to MSCs-*TetR*/*Amelx* on day 0–10 (**a**: early stage), day 10–20 (**b**: intermediate stage), or day 20–30 (**c**: late stage) of osteogenic differentiation, and Alizarin Red S staining was performed at day 10, day 20, or day 30, respectively. The staining intensity was also quantitatively analyzed. Significant differences (*P<0.01: ANOVA with Tukey’s multiple comparison test: n = 9) are shown.

Alizarin Red S staining showed that addition of Dox at the early stage of osteogenic differentiation significantly induced matrix calcification of MSCs-*TetR*/*Amelx* on day 10 (Fig 6a). When Dox was applied to MSCs-*TetR*/*Amelx* on day 10–20 (intermediate stage) or day 20–30 (late stage) of osteogenic differentiation, matrix calcification of the MSCs-*TetR*/*Amelx* was not significantly altered (Fig 6b and 6c).

## Discussion

The amelogenin gene sequence is present in both the human X and Y chromosomes, whereas in the mouse it is found exclusively in the X chromosome [26]. Therefore, production of amelogenin protein in mice is encoded by the *Amelx* (*amelogenin*, *X-linked*) gene. In this study, we successfully established a Tet-controlled *Amelx* gene regulation system for mouse MSCs in which transcriptional activation of *Amelx* was associated with enhanced osteogenic differentiation.

Other than the Tet-dependent transcriptional regulatory system, recombinant adenoviruses that express site-specific recombinases such as Cre and FLP are widely used to provide a molecular switch to turn on/off transgene expression in cultured mammalian cells [27]. Although this system is attractive in that it shows little or no leakage of the target gene, control (on/off) of the target gene is accomplished by “transient” expression of the adenoviral recombinase; therefore, the duration of period in which gene expression can be controlled could be limited. In addition, recombinant adenovirus systems require transduction of adenovirus recombinase at each on/off regulation step, which is expected to be labor-intensive and time-consuming. In contrast, the Tet-dependent regulatory system has the advantage of maintaining the on/off regulation for a desired period of time, without limitation, simply by addition or removal of Dox.

It is known that primary mBMSC populations contain high proportions of non-MSCs and hematopoietic cells [28,29], which may lead to uneven and unpredictable behavior of individual cells during establishment of the Tet-dependent regulatory system. Therefore, in this study we used a clonal and immortalized population of MSCs (mBMSC-4 line) [20]. This cell line is multipotent, as demonstrated by its ability to differentiate specifically into osteoblast [30], chondrocyte [31], adipocyte, and myoblast [20] lineages and also to form ectopic bone *in vivo* [32].

According to the manufacturer’s information, the *TetR* expression vector and TO/target gene expression vector can be introduced into cells at the same time; however, in the present study we first established *TetR*-expressing MSCs, followed by transduction of the pLenti6.3/TO/V5/*Amelx* expression vector. The advantage of our method is that the established MSCs-*TetR* can be used for Tet regulation of not only *Amelx* but also any other target gene in future experiments. The expression vectors for such genes can be easily prepared because our system utilizes the Gateway cloning system as a template.

The established MSCs-*TetR*/*Amelx* enhanced expression of the *Amelx* gene and amelogenin protein in the presence of Dox. Although slight leakage of *Amelx* gene expression without Dox induction was detected by RT-PCR, there was no background expression of amelogenin protein when the cells were cultured in the absence of Dox. Many studies have indicated that leakage of transgene transcription in Tet-dependent regulatory systems is unavoidable and acceptable in most experimental cases [33,34]. Because MSCs-*TetR* and MSCs-*TetR*/*Amelx* were established from single colonies during the drug selection process, MSCs-*TetR*/*Amelx* appeared to be homogeneous. Indeed, the enhanced *Amelx*-induced calcification in MSCs-*TetR*/*Amelx* at early passages (Fig 5c and 5d) was reproducible in those cells after repeated passaging (Fig 6a), indicating that MSCs-*TetR*/*Amelx* maintained their homogeneity with osteogenic activity even after repeated passaging.

In this study, we focused on the effects of forced expression of *Amelx* on MSCs that were undergoing osteogenic differentiation. Hu *et al*. [16] evaluated the genome-wide expression profile of human MSCs without induced differentiation after lentiviral overexpression of amelogenin. In their approach, permanent expression of amelogenin should have been initiated immediately after lentiviral transduction, and undesired effects of the uncontrolled exogenous amelogenin expression may have occurred during drug selection. In this regard, the controllable expression system established in our study has the advantage of controlled initiation or cessation of amelogenin expression at desired time points, which we specifically utilized in this study of the time-dependent osteogenic differentiation of MSCs. Expression of *Amelx* in MSCs-*TetR*/*Amelx* was obviously enhanced or reduced by addition or removal of Dox during osteogenic differentiation. These results indicate that we successfully established a Tet-controlled *Amelx* gene regulation system for MSCs, in which the expression of *Amelx* could be regulated by Dox addition and removal even during the differentiation process.

Amelogenin has cell signaling properties [35–38]. We thus examined whether the controllable expression of *Amelx* would concomitantly affect the expression of these osteogenic genes. Interestingly, expression of *osterix*, *BSP*, *osteopontin* and *osteocalcin* was altered in parallel with the controlled expression of *Amelx*. In particular, *BSP* and *osteopontin* were extensively up-regulated by *Amelx* expression, implying that these phosphorylated sialoglycoproteins may be preferential target molecules for *Amelx* signaling during osteogenic differentiation of MSCs. Shimizu *et al*. demonstrated that amelogenin stimulates *BSP* expression in osteoblasts through the fibroblast growth factor 2 (FGF2) response element and transforming growth factor-β1 (TGF-β1) activation element in the promoter of the *BSP* gene [36]. Amelogenin promotes osteogenic differentiation of MSCs through the Wnt/beta-catenin signaling pathway by up-regulating *Wnt10b* [37]. Olivares-Navarrete *et al*. recently reported that both amelogenin and N-terminal amelogenin peptide (NTAP) induced osteogenic differentiation of human MSCs, and that the effects of the NTAP were mediated through PKC and ERK1/2 activation and β-catenin degradation [38]. In this study, the concomitant expression of *osterix*, *BSP*, *osteopontin*, and *osteocalcin* with the controlled expression of *Amelx* implies that the exogenous *Amelx* expression affects the transcriptional activation of these genes. In contrast, *Runx2* expression was not significantly affected by the controlled expression of *Amelx*. Although *Runx2* is a crucial transcriptional factor for osteoblast differentiation, osterix exerts its osteogenic function via *Runx2*-independent mechanisms [39–41]. The controlled expression of *Amelx* in our study may therefore have regulated *osterix* via *Runx2*-independent pathways, which in turn promoted expression of other osteogenic genes. However, our work appears to contradict recently published studies showing that recombinant amelogenin and its peptides promote expression of *Runx2* mRNA in MSCs [37,38]. This discrepancy may have resulted from differences in the application of amelogenin, i.e., extracellular administration of amelogenin proteins/peptides vs. intracellular expression of *Amelx* mRNA. Further studies are necessary to elucidate these signaling pathways.

Osteogenic differentiation of MSCs is a well-orchestrated process that begins with activation of transcription factors including *Runx2* and *osterix* [42]. In the late stage of the osteoblast developmental sequence, *BSP*, *osteopontin*, and *osteocalcin* serve as regulators of the mineralization process [43]. Many studies have shown that enamel matrix derivative (EMD), the active component of Emdogain that contains heterogeneous growth factors including amelogenin, stimulates mineralizing cell types to increase their ALP activity and production of osteopontin, BSP, and osteocalcin [44]. However, previous reports on the effects of recombinant amelogenin on osteogenic differentiation have presented relatively contradictory results. Matsuzawa *et al*. reported that mouse recombinant amelogenin increased *type I collagen* and *osteocalcin* mRNA levels in an osteoblast cell line (ROS17/2.8 cells) [45]. Zeichner-David *et al*. reported that mouse recombinant amelogenin induced expression of *BSP* and *osteocalcin* but down-regulated *type I collagen* in mouse periodontal ligament cells. In mouse cementoblasts, recombinant amelogenin and tyrosine-rich amelogenin peptide were shown to down-regulate *BSP* and *osteocalcin* and inhibit mineral nodule formation [11,46]. Therefore, the effects of amelogenin on osteogenic differentiation may depend on the cell type and on whether full-length or domain-derived peptides are used.

Regarding the effects of amelogenin on MSCs, increased osteogenesis was previously reported after treatment with recombinant amelogenin [15,38] or NTAP [38]. However, no data are available yet for the effects of forced expression of *Amelx* on MSCs under osteogenic guidance, especially for matrix mineralization of MSCs. In this study, MSCs-*TetR*/*Amelx* treated with Dox during osteogenic induction showed increased expression of the osteogenic marker genes *osterix*, *BSP*, *type I collagen* and *osteocalcin*. The up-regulation of these osteogenic genes resulted from expression of exogenous *Amelx* and not from a direct effect of Dox because Dox treatment did not significantly alter osteogenic gene expression in non-transduced MSCs (data not shown). Osterix is a typical transcription factor required for osteoblast differentiation and bone formation [47]. Type I collagen is a primary product of osteoblasts, and its gene expression increases at the early to intermediate stages and decreases gradually thereafter during bone matrix formation [48]. Expression of *BSP* is detected in more extensively differentiated osteoblasts, i.e., at a relatively late stage of differentiation [49]. BSP is a non-collagenous protein component of mineralized tissues, such as cementum and bone, and is believed to be a critical molecule for promoting biomineralization [49,50]. Thus, given the roles of these molecules in osteogenesis, their up-regulation by *Amelx* transduction may have contributed to the observed enhancement of MSC osteogenic differentiation. However, forced expression of *Amelx* alone appears to not be sufficient to induce osteogenic differentiation because Dox did not significantly stimulate osteogenic marker gene expression (Fig 4b–4d) under the non-induction condition. In addition, forced expression of *Amelx* appears to not induce dentin marker expression in MSCs under osteogenic induction because expression of DMP1 and DPSS in MSCs-*TetR*/*Amelx* was not stimulated by the presence of Dox.

Concomitantly with the up-regulation of osteogenic genes by transcriptional activation of *Amelx*, the ALP activity of MSCs-*TetR*/*Amelx* also clearly increased, and Dox treatment enhanced the matrix calcification of MSCs-*TetR*/*Amelx* in the osteogenic induction medium. These results provide further evidence that transcriptional activation of *Amelx* enhances the osteogenic differentiation of MSCs. However, the 2.5- (*osterix* and *type I collagen*) and 5.5- (*BSP*) fold increase in the expression of these genes (Fig 4b–4d) may not be sufficient to aggressively impart a mature osteoblastic phenotype to MSCs. Indeed, the matrix calcification of the MSCs was already significantly increased on day 10 after *Amelx* transduction (Fig 6a), although expression of the osteogenic marker genes did not markedly increase until day 7 (Fig 4a–4d). Full-length amelogenin has the capacity to stabilize the formation of amorphous calcium phosphate (ACP) [51], and during bone biomineralization, an abundant ACP phase serves as a precursor phase that later transforms into mature crystalline calcium phosphate [52,53]. Deshpande *et al*. recently demonstrated that amelogenin interacts with collagen fibrils and mineral particles to mineralize the collagen fibrils [54]. Therefore, the increased ECM mineralization observed after *Amelx* transduction may involve direct effects of the expressed amelogenin protein on ECM mineralization in addition to the effects mediated through up-regulation of osteogenic genes. The direct regulation of calcium phosphate mineral formation by amelogenin would thus be expected to mainly contribute to the precursor phase of biomineralization, which would explain why forced expression of *Amelx* initiated in the intermediate and late stages of the osteogenic differentiation did not significantly contribute to MSC calcification (Fig 6b and 6c). Information from the present study and the continued study of Tet-dependent *Amelx* regulatory systems in MSCs may help to clarify the mechanisms by which amelogenin regulates key molecules associated with bone mineralization.

## Conclusions

This study established a Tet-controlled *Amelx* gene regulation system for MSCs that could be a beneficial tool to investigate novel functions of amelogenin in MSC osteogenesis. The present data show that transcriptional activation of *Amelx* enhances osteogenic differentiation of MSCs *in vitro* by up-regulating *osterix*, *BSP*, *osteocalcin* and *type I collagen*. In particular, *Amelx* expression was suggested to affect the osteogenic differentiation of MSCs through cell signaling roles associated with expression of *osterix*, *BSP*, *osteopontin*, and *osteocalcin*. Forced *Amelx* expression was also suggested to enhance ECM mineralization through a direct effect of amelogenin on calcium phosphate mineral formation at the early stage of biomineralization. These findings represent an important step toward the optimal application of amelogenin therapy for periodontal/bone regeneration.
